# Editorial: Recent advances and future prospects for sustaining a healthier food system

**DOI:** 10.3389/fnut.2025.1696614

**Published:** 2025-10-14

**Authors:** Monika Thakur, Ana Sanches Silva, Karuna Singh, Vinod Modi

**Affiliations:** ^1^Amity Institute of Food Technology, Amity University Uttar Pradesh, Noida, India; ^2^Faculty of Pharmacy, University of Coimbra, Coimbra, Portugal; ^3^Centre for Animal Science Studies (CECA), Instituto de Ciências, Tecnologias e Agroambiente, University of Porto, Porto, Portugal; ^4^Associate Laboratory for Animal and Veterinary Sciences (Al4AnimalS), Lisbon, Portugal; ^5^Sharda School of Allied Sciences, Sharda University, Greater Noida, Uttar Pradesh, India

**Keywords:** SDG's, healthier food system, novel foods, food safety, sustainable agriculture, nutritional strategies

## Background of global food systems

Over the past 50 years, the Global food systems have changed from being diverse and rural to being highly industrialized and concentrated, with significant effects on environmental resilience, nutrition, and livelihoods. Despite advancements, problems like food insecurity, obesity, malnutrition and hidden hunger still exist and portend impending public health crises. As countries work to accomplish the Sustainable Development Goals (SDGs) while tackling deeply rooted and interconnected problems like hunger, malnutrition, climate change, inequality, biodiversity loss, rising food prices, and food insecurity in conflict-affected areas, the transformation of food systems has become a key challenge for the 21st century. Innovations in sustainable food production, food processing, and nutritional sciences are all necessary to create robust, healthier food systems. Global health outcomes are still at risk due to persistent malnutrition, food instability, obesity, and nutritional deficiencies. Achieving the Sustainable Development Goals (SDGs) requires creating food systems that are more resilient, equitable, and healthful (1, 2).

## Introduction

Agri-food systems worldwide face mounting pressure from rising demand, malnutrition in all its forms, climate change, overexploitation of resources, biodiversity loss, and persistent food waste. These threats compromise the capacity of nations to ensure adequate and nutritious food for present and future generations (3). Against this backdrop, the contributions published in this Research Topic emphasize not only technological advances but also integrative strategies linking health, sustainability, and equity. Recent Advances in Healthier and Sustainable Food Systems includes various major concerns as—Alternative Foods and Protein Sources, Cutting-Edge Food Technologies, Food Safety, Waste Valorization, and Circularity, Consumer Health, Nutritional Design, and Policy Frameworks.

## Recent findings

The most recent findings on the theme have been further elaborated by the different researchers as:

- Ma and Zhang examine *deep-sea aquaculture* as a promising strategy to ensure sustainable aquatic food supplies while meeting growing global nutritional demands. Using an evolutionary game-theory framework, they explore the optimum role of government in fostering this industry. They conclude that while government leadership is essential in early stages, its role should shift to guidance as the industry matures. Key to sustainable adoption is a fair, risk-balanced structure where total risk and costs remain lower than combined benefits and subsidies, and effective partnerships between businesses and supporting organizations are established.
- Wakayama et al., in their study “*Development and Validation of the Meiji Nutritional Profiling System for Children,”* introduce a rigorously validated, age-specific framework—namely the Meiji Nutritional Profiling System (Meiji NPS)—designed to assess the nutritional quality of foods for children aged 3–11 years. By calibrating the system to distinguish between younger (3–5) and older (6–11) age groups and demonstrating strong correlation (*r* = 0.73) with established indices like the Nutrient-Rich Foods Index 9.3, the study underscores the value of tailored, transparent, evidence-based profiling tools in guiding healthier dietary choices and product reformulation for younger populations.
- Mehta and Bhattacharjee, in their study “*Sustainable Pork Production and Processing: A Step Toward Empowering Tribal Women in Northeast India,”* investigate how sustainable pork production and processing can serve as a catalyst for tribal women's economic empowerment in the Northeastern Region (NER) of India—an area marked by poverty, gender disparity, and limited livelihood options. The authors advocate for targeted policy support and the cultivation of supportive networks to scale sustainable pork production systems across the NER. The findings from the post-impact analysis of the capacity-building approach call for policy intervention and the establishment of supportive networks to enhance the growth of a sustainable pork production system across NER, thereby contributing to the attainment of Sustainable Development Goal (SDG) targets proposed by the Indian economy.
- Gerber and Roberts in their narrative review “*Peanut Hulls, an Underutilized Nutritious Culinary Ingredient: Valorizing Food Waste for Global Food, Health, and Farm Economies”*—highlight the overlooked value of peanut hulls as a promising source of nutrition and sustainability. Though traditionally relegated to livestock feed or construction materials, peanut hulls contain substantial amounts of dietary fiber, protein, and phytonutrients such as phenols (e.g., luteolin and resveratrol). Their review consolidates evidence supporting the safe incorporation of these hulls into widely consumed products—like bread or plant-based meat alternatives—to improve nutrient density, promote human health, and reduce food waste, all within a Food Systems Approach (FSA) aligned with multiple UN Sustainable Development Goals. The study also emphasizes the untapped economic benefits for farmers and the potential of emerging green processing technologies to enable future commercialization of peanut hulls for human consumption, despite current lack of industrial-scale operations.

This diversity of research exemplifies the integrative nature of food systems science—addressing challenges across production, consumption, and policy while promoting biodiversity, health, and sustainability.

## Recent advances and future prospects in formatting a healthier food system

The Research Topic entitled “*Recent Advances and Future Prospects in Formatting a Healthier Food System”* presents a comprehensive collection of original research and reviews encompassing novel foods, food safety, sustainable agriculture, and nutritional strategies. Readers will recognize the breadth of challenges addressed and the new opportunities explored in building healthier, sustainable food systems. These contributions align with the SDGs and underscore the pivotal role of science, technology, and innovation in advancing nutrition security, environmental stewardship, and overall wellbeing. These contributions underscore the vast landscape of challenges addressed—from food security and waste reduction to cutting-edge technological interventions and highlight pathways toward building healthier, more sustainable food systems.

**Figure 1:**
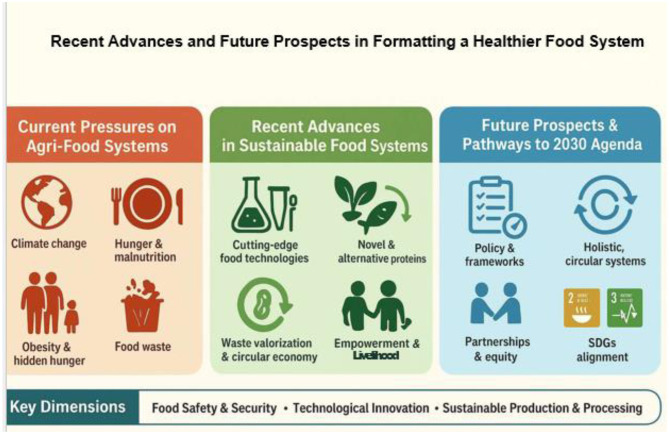
Recent advances and future prospects of sustainable food systems.

The Research Topic outlines specific goals that guide its vision and scope (Figure 1). These include:

- Food Safety, Security, and Healthy Diets
- Technological Innovation & Novel Foods
- Production & Processing Strategies
- Strengthening Healthier Food Systems through R&D

The objectives of the Research Topic closely resonate with three pivotal dimensions shaping the evolution of global food systems:

- Holistic, Circular Systems
- Technological Drivers of Sustainability
- Policy & Multidisciplinary Integration

## Conclusion

Therefore, understanding the challenges and opportunities in food system transformation is vital because it directly impacts our ability to achieve the SDGs, particularly those related to Zero Hunger, Good Health and Wellbeing, and Climate Action. By harnessing innovation and research in this field, we can work toward creating more efficient, sustainable, and equitable food systems, thereby advancing the broader global goals of Agenda 2030.
